# Absence of human rhinovirus and respiratory syncytial virus from bronchoalveolar lavage and bronchial biopsies of selected patients with stable chronic obstructive pulmonary disease

**DOI:** 10.1186/s12931-016-0323-x

**Published:** 2016-01-27

**Authors:** Styliani Giannakaki, Lida Politi, Elvira Markella Antonogiannaki, Nick Spanakis, Georgios Arsenis, Vasiliki Filaditaki, Spyridon Zakynthinos, Napoleon Karagiannidis, Athanassios Tsakris

**Affiliations:** 2nd Respiratory Medicine Department, Sismanogleio-A.Fleming General Hospital, Athens, Greece; Department of Microbiology, Medical School, University of Athens, Athens, Greece; 1st Department of Critical Care and Pulmonary Services, Evangelismos Hospital, Medical School, University of Athens, Athens, Greece

**Keywords:** Stable COPD, Bronchoalveolar lavage, Biopsy, HRV, RSV, Real-time PCR

## Abstract

Previous studies have reported very different rates of human rhinovirus (HRV) and respiratory syncytial virus (RSV) genome detection in nasal and sputum samples, but not in bronchoalveolar lavage (BAL) and bronchial biopsy samples. Our study aimed to investigate the presence of HRV and RSV in the lungs of 31 consecutive patients with stable COPD (11 GOLD stage I, 11 II, and 9 III) and 22 control subjects (12 current or past smokers, and 10 non-smokers), who underwent diagnostic (e.g., lung cancer) and/or therapeutic (e.g., hemoptysis) fibreoptic bronchoscopy in a university hospital in Athens, Greece. Viral RNA of HRV and RSV were not detected in any of the samples of COPD patients or control subjects after being processed with real-time PCR.

## Findings

### Background

The potential role of persistent low-grade, latent viral infection in disease progression in stable COPD is a matter of great importance [1, 2] and controversy partly due to differences in the reported occurrence of asymptomatic virus infection in COPD. Indeed, in the study by Seemungal et al. [3] HRV and RSV were detected in blood samples/nasal aspirates in 7,4 and 23,5 % of patients with stable COPD, respectively, whereas in the study by Falsey et al [4] nasal and sputum samples were positive for RSV in only 0 and 0,9 %, respectively. Importantly, none of the previous studies evaluated samples coming directly from the lower airways, i.e., bronchoalveolar lavage (BAL) fluid or bronchial biopsies, which are fully representative of the lower respiratory tract. Nevertheless, it is constantly stressed out that further studies across the spectrum of disease as well as in smoking and non smoking control subjects are required to better understand the role of asymptomatic viral infection in stable COPD [1, 2].

Therefore, the aim of this study was to investigate whether HRV and RSV are present in the lungs of stable COPD patients by performing BAL and bronchial biopsies, and relate their presence with disease severity.

## Methods

The population of the study consisted of consecutive patients with stable COPD and control subjects (non-smokers or current/ex-smokers), who were referred to the Sismanoglion Hospital, Athens, Greece, between October 2012 and November 2014 in order to undergo fibreoptic bronchoscopy for diagnostic (e.g., lung cancer) or therapeutic (e.g., hemoptysis) purposes. At study time, the clinical and functional status of COPD patients had been stable for at least 4 weeks [5], and none had received antibiotic treatment during the last 4 weeks. Exclusion criteria included atopic history, asthma, extensive pleural effusions, bronchiectasis, immunosuppression due to chemotherapy or systemic corticosteroids and all the contraindications of the bronchoscopic procedures.

All the participants completed a questionnaire which included demographic characteristics, cigarette smoking status (pack/year) and their past medical history. Τheir clinical status was evaluated according to the modified Medical Research Council (mMRC) scale of breathlessness and the Body mass, airflow Obstruction, Dyspnea, Exercise (BODE) index. Pulmonary function testing was performed, and arterial blood gases and blood oxygen saturation by pulse oximetry in the sitting position were measured. We performed BAL from subsegmental bronchi and bronchial biopsies (3–4 per patient) from non infiltrated carina. RNA samples were extracted from BAL and lung biopsies and a nucleocapsid (N) gene partial sequence, as well as a 5’ non-coding region (5΄NCR) partial sequence were amplified for RSV and HRV, respectively. Real-time PCR studies were performed in Stratagene Mx3005 Real-Time PCR Instrument (Agilent Technologies, Santa Clara, CA, USA). The primer and probe mix provided exploited the so-called TaqMan principle. Each time the kits were used, a positive and a negative control reaction were included in the run.

The study was approved by the local Medical Ethics Committee of Sismanoglion Hospital and all subjects gave their informed consent.

## Results

We enrolled 31 patients with COPD and 22 control subjects. The most common symptoms were dyspnea (32 %), cough (19 %) and hemoptysis (13 %). Whereas 47 % of participants did not mention any symptom, but referred to our department in order to investigate bronchoscopically abnormal radiographic findings revealed in chest computed tomography. We diagnosed lung cancer in 63,6 % of control subjects (adenocarcinoma: 4, squamous cell carcinoma: 9, small cell carcinoma:1), and in 67,7 % of COPD patients (adenocarcinoma:7, squamous cell carcinoma: 8, small cell carcinoma: 6). The commonest comorbidities of COPD patients were arterial hypertension (47 %), hyperlipidemia (20.7 %) and coronary artery disease (17 %), and of control subjects arterial hypertension (50 %), hyperlipidemia (22,7 %) and diabetes mellitus type 2 (13,6 %).

Anthropometric characteristics and lung function data of both COPD patients and control subjects are shown in Table 1. Controls and COPD patients had comparable age, FVC, D_LCO_ and PaCO_2_. As expected, COPD patients had lower spirometric values, arterial blood gases measurements and higher rate of smoking habits, mMRC scale and BODE index scores compared to control subjects.

**Table 1:** Anthropometric characteristics and respiratory function data of COPD patients and control subjects

Parameters	COPD	Controls
	(*n* = 31)	(*n* = 22)
Gender, Males (%)	26 (83,8 %)	16 (72,7 %)
Age, years	67.6 ± 8.1	62.4 ± 11.7
BMI, kg/m^2^	25.3 ± 4.6**	25.7 ± 4.5
Pack/Years	57 ± 27*	35.45 ± 35
FVC, % pred	85.6 ± 20.1	93.6 ± 15.9
FEV_1_, % pred	70.8 ± 18.6***	93.3 ± 15.9
FEV_1_/FVC, %	63.7 ± 8.04***	78.9 ± 5.28
Spirometric classification of COPD: I, II, III, IV	11,11,9,0	NA
PEF, % pred	69.4 ± 20*	87 ± 23
DL_CO_, % pred	72.1 ± 25.5	85 ± 22.8
SpO_2_, %	94.7 ± 3.2*	96.45 ± 1.2
PaO_2_, mmHg	70.2 ± 12.1*	79.57 ± 6.47
PaCO_2_, mmHg	38 ± 2.25	40.1 ± 1.7
mMRC scale (0,1,2,3,4)	1.09 ± 1** (10,13,5,1,2)	0.36 ± 0.72 (16,5,0,1,0)
Symptom/risk evaluation of COPD: A, B, C, D	19,3,4,5	NA
BODE index (0-2,3-4,5-6,7-10)	1.96 ± 2.37** (23,4,2,2)	0.4 ± 0.73 (21,1,0,0)
Inhaled steroids, n (%)	4(7.5 %)	0(0)

Values are means (±SD)

Abbreviations: *BMI* body mass index; *FVC* forced vital capacity; *FEV*_*1*_ forced expiratory volume in one second; *NA* Non Applicable; *PEF* peak expiratory flow; *DL*_*CO*_ diffusing capacity of the lung for carbon monoxide; *SpO*_*2*_ % arterial oxygen saturation measured with pulse arterial oximeter; *PaO*_*2*_ partial arterial oxygen tension (at room air); *PaCO*_*2*_ partial arterial carbon dioxide tension; *mMRC* modified Medical Research Council scale (0: not troubled by breathlessness except on strenuous exercise, 1: shortness of breath when hurrying on the level or walking up a slight hill, 2: walks slower than people of the same age on the level because of breathlessness or has to stop for breath when walking at own pace on the level, 3: stops for breath after walking about 100 m or after a few minutes on the level, 4: too breathless to leave the house or breathless when dressing or undressing); Symptom/risk COPD evaluation: A = Low risk-Less symptoms/B = Low risk-More symptoms/C = High risk-Less symptoms/D = High risk-More symptoms; BODE index: Body mass index (kg/m^2^), airflow Obstruction (FEV_1_% pred), Dyspnea (mMRC scale), Exercise (6 min walking distance) (0–2 points: 80 % 4 year survival, 3–4: 67 %, 5–6: 57 %, 7–10: 18 %)

*p < 0.05 compared with controls; **p < 0.01 compared with controls; ***p < 0.001 compared with controls

**Fig. 1:**
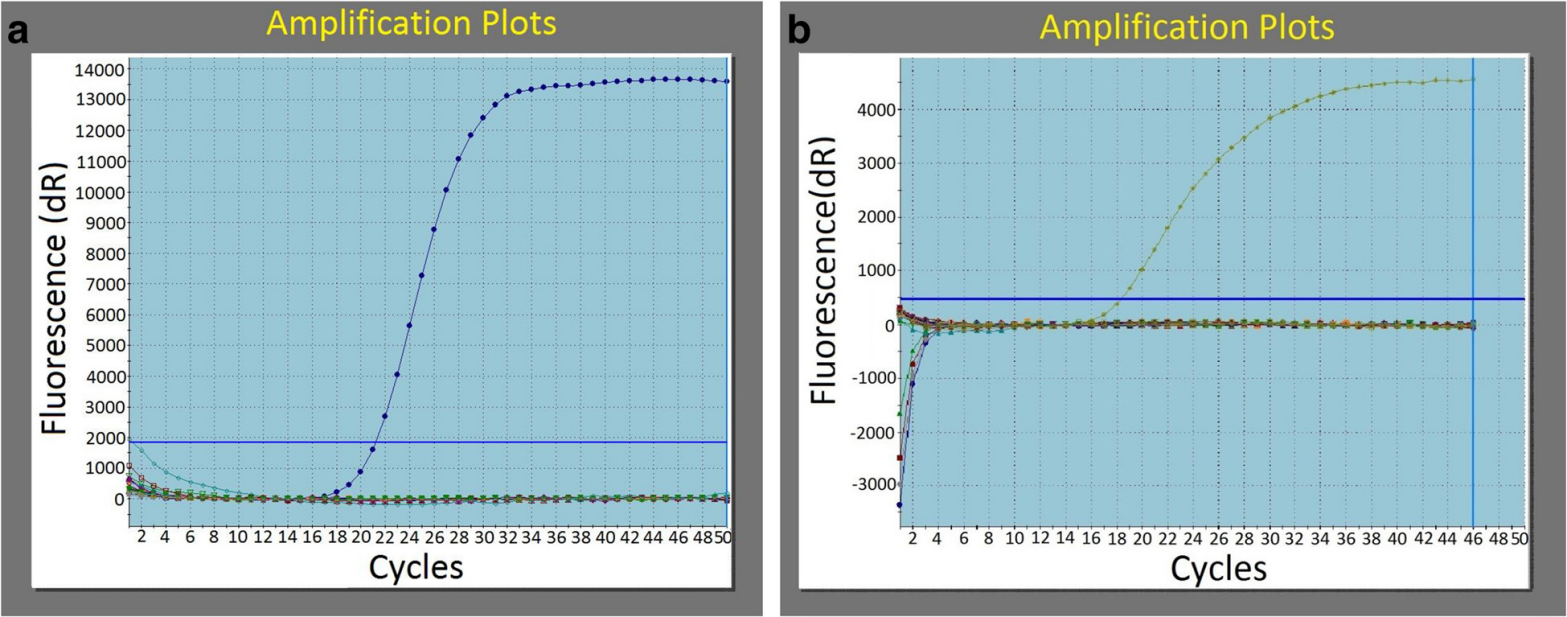
**a** Representative amplification plot for HRV. Horizontal blue line: baseline of the method. Curved blue line: positive control. Remaining lines: negative controls and samples. **b** Representative amplification plot for RSV. Horizontal blue line: baseline of the method. Curved blue line: positive control. Remaining lines: negative controls and samples

All BAL and bronchial biopsy samples (53 and 181, respectively) were processed for the detection of HRV and RSV genome with real-time PCR. In Fig.1a and b we can see representative amplification plots for HRV and RSV, respectively.

In all amplification plots for HRV and RSV, samples’ and negative controls’ fluorescence signals were at the same level indicating that these two viruses were not detected in any of the samples of COPD patients and control subjects.

## Discussion

There are sparse publications and controversial reports concerning viral infection of the respiratory tract in stable COPD [1, 3, 4, 6]. Our study had several limitations that probably influenced the results. More specifically, we included patients from 3 of the 4 GOLD stages as it was rather difficult to perform bronchoscopy in patients with very severe COPD. This could have an impact on our results as the relative importance of airway bacterial infection in COPD has been shown to increase with disease severity [7]. Also, most of the COPD patients did not experience any exacerbations during the year before recruitment, while there is a tendency for patients in whom HRV and RSV are detected in stable COPD to give a history of more frequent exacerbations in the previous year [3]. Additionally, the number of participants in the study was relatively small, and the enrollment of patients was performed in only one center.

On the other hand, the detection rates for HRV and RSV among the two groups of normal control subjects and the results were zero, which is similar to previous reports [8, 9]. In addition to this, the majority of our participants did not use inhaled and oral corticosteroids on a chronic basis. This is very important as prolonged shedding of RSV has been described in a number of immunosuppressive conditions such as corticosteroid use and HIV infection [10]. The PCR kits that we used are designed to have the broadest detection profile possible whilst remaining specific to the HRVsp and to the RSV_spp genome. We ensured that the samples were suitable in terms of purity, concentration, and RNA/DNA integrity and we always run at least one negative control with the samples. An alternative explanation for the discrepancy between this study and previous reports is the possibility of PCR contamination which leads to high rates of HRV and RSV RNA identification in subjects with stable COPD.

In conclusion, we have shown for the first time using bronchoscopy that both HRV and RSV RNA were not detected in the BAL and bronchial biopsy samples collected from patients with stable COPD and from control subjects. The possibility of chronic HRV and RSV infection as a mechanism of disease progression could have enormous impact on the management of the disease but our study does not support this hypothesis.

## Abbreviations

BAL: Bronchoalveolar lavage
BMI: Body mass index
BODE: Body mass index, airflow obstruction, dyspnea, exercise
COPD: Chronic obstructive pulmonary disease
DL_co_: Diffusing capacity of the lung for carbon monoxide
FEV_1_: Forced expiratory volume in the first second
FiO_2_: Fraction of inspired oxygen
FVC: Forced vital capacity
GOLD: Global initiative for chronic obstructive lung disease
HRV: Human rhinovirus
mMRC scale: modified Medical Research Council scale
PaCO_2_: Partial arterial carbon dioxide tension
PaO_2_: Partial arterial oxygen tension
PEF: Peak expiratory flow
RSV: Respiratory Syncytial Virus
SPO_2_ %: Arterial oxygen saturation measured with pulse arterial oximeter
